# Traditional healing for physical and mental problems in the Arab region: past and current practices

**DOI:** 10.1192/bji.2024.3

**Published:** 2024-05

**Authors:** Brigitte Khoury, Mira Rafeh, Zeina Bou Dargham

**Affiliations:** 1 Professor, Department of Psychiatry, American University of Beirut, Beirut, Lebanon Email: bk03@aub.edu.lb; 2 Intern, Department of Psychiatry, American University of Beirut, Beirut, Lebanon; 3 Intern, Department of Psychiatry, American University of Beirut, Beirut, Lebanon

**Keywords:** Traditional medicine, healing, spiritual healing, transcultural psychiatry, community mental health teams

## Abstract

Exploring traditional healing practices in the Arab world unveils a diverse range of methods deeply rooted in ancient beliefs. Traditional healing practices encompass natural remedies, spiritual rituals and physical treatments. These historical practices persist today, reflecting their enduring relevance in Arab culture and their influence on healthcare approaches. Factors such as accessibility to traditional healing services, a lack of affordable medical treatment, cultural familiarity and a strong belief in the efficacy of traditional healing methods in treating mental problems contribute to their continued use. However, potential challenges arise when an exclusive reliance on traditional methods might hinder access to critical medical interventions. Thus, the need for further documentation and research into these deeply ingrained healing traditions is emphasised. Some research has focused on integrating these traditional approaches with the modern medical system, recognising their combined value in healthcare. This balanced approach holds the potential to bridge the gap between culturally informed traditional practices and contemporary medical treatments.

The World Health Organization defines traditional medicine as the totality of knowledge, skills and practices based on culturally specific theories, beliefs and experiences.^1^ Whether or not they align with the modern medical model, these practices are still used for the preservation of health as well as the prevention, diagnosis, improvement or treatment of physical and mental illness via non-medical means.^2^ Natural remedies, including those derived from plants, animals and minerals, as well as spiritual, exercise, acupuncture and massage treatments, are all included in traditional healing techniques. The terms ‘complementary’ and ‘alternative’ medicine are also used to describe traditional medicine, referring to the wide variety of medical techniques that are not part of a nation's predominant medical system.^1^

## History of traditional healing in the Arab region

The Arab region has a rich history of medicine, evidenced by ancient written documents on health-related beliefs, practices and professional qualifications.^3^ Additionally, these historical documents reveal that prevalent healing practices in Middle Eastern nations are firmly embedded in healing traditions such as Pharaonic, Hellenic, Sufi, Prophetic or Islamic, and Christian traditions, all of which emerged to prominence before the arrival of Western medicine in the area during the 19th-century colonial period.^3,4^ Prior traditions continue to exist – not as integrated healthcare systems, but as a diverse array of aetiological, diagnostic and therapeutic beliefs and practices about the origins of wellness and diseases.^5^

These traditional healing practices date back to the 7th century ad when Islam first emerged, when the focus was shifted towards soul and bodily healing methods that considered both prevention and therapy.^6^ Prophetic medicine has historically been well-liked by the great majority of people since it adopted popular ideas and techniques from Islamic theology, such as etching religious verses on healing charms, the concept of the evil eye and the application of heated cups to the skin. All of these practices are still widely used in many Middle Eastern countries today.^6^ In addition, ‘Sufi’ groups were considered to be popular Islamic mystic sects that served the spiritual, psychological, medical and political demands of the poorer classes.^3^ Their offered medical expertise ranged from treating psychological conditions that are usually associated with issues such as spirit possession to dealing with female health problems and fertility. The tradition of prophecy and Ṣūfī healing continues to thrive in many parts of the Middle East, especially in North African countries. Large numbers of Muslim believers still visit holy sites in cities and rural places across the Arab world, in countries such as Morocco, Tunisia and Egypt, for divine blessings, holiness or grace.^5^

Similar references to medicine and healing practices have been highly prevalent among Christian communities throughout history until modernity. References to the magical, miraculous and spiritual healing of illnesses involve the role of divine intervention.^7^ For instance, Saint Charbel was hailed as the ‘Doctor of Heavens’, with miracles of healing the sick. Christians in the Levant and nearby areas continue to travel to visit the holy site of Saint Charbel convent, in quest of divine blessings, heavenly graces and protection, especially when there is illness in the family. Additionally, widespread conventional healing practices have been performed in Christianity, such as exorcism. Exorcism has been historically utilised by ‘prehistoric shamans, witch doctors, priests, and medicine men’ to help in treating possession by demonic spirits and reducing its impact on associated symptoms.^8^ This had gained controversy with increased education on mental illnesses, and the uncovered overlap between the symptoms of mental illness and spirit possession.

## Traditional versus Western interventions

Following the incursion of the ‘Western’ biomedical model into Arab and Muslim countries, Islamist intellectuals proposed ‘Islamic medicine’ as a countermeasure, driven by a broader political agenda aimed at avoiding the Westernisation of Muslim societies.^4^ Islamic medicine opposed the biomedical model of illness for disregarding the spiritual side of people. Several herbal cures were encouraged at the time, in addition to ‘traditional’ theories and faith healing through prayer and the recital of sacred texts. Herbal usage has persisted throughout time and is currently prevalent in the Arab world. A recent research study involving hospital out-patients in Saudi Arabia found that 76% acknowledged using herbal medication, indicating that the consumption of herbal medicine is high both globally and in Saudi Arabia specifically.^9^

Although the majority of such interventions have been shown to be helpful, if they involve delaying or preventing prompt access to modern medical healthcare, they could cause potential damage to patients.^4^ A systematic review conducted in the Arab world, covering countries such as Egypt, Saudi Arabia, Morocco, Iraq and Jordan, revealed that a significant number of patients with mental disorders initially turn to faith healers for mental healthcare, without seeking any other form of medical care.^10^ Ultimately, placing sole dependence on faith and spiritually based services might impede efforts to seek medical attention or disease prevention and slowing the progression of illnesses.

Statistics have also shown that around 80% of the population living in rural areas of low- and middle-income countries depend on traditional medicine for their mental health needs.^1^ The prevalence of traditional healing services in these areas may be attributed to multiple factors: accessibility, a lack of affordable medical care and a strong belief in the efficacy of traditional healing methods for mental disorders. This is exemplified in a recent Sudanese study, in which 89% of participants sought traditional medicine, specifically medical herbs and holy recitation.^11^ The study affirmed that affordability, cultural and religious influences and perceived effectiveness were pivotal motivations driving individuals to seek traditional treatments. Additionally, the prevalence and pattern of traditional healing service usage are significantly influenced by political and situational contexts. This influence was notably exemplified in reports from local news platforms in war-torn Yemen.^12^ Escalating prices of prescription drugs, soaring medical expenses and the collapse of the healthcare system have compelled civilians to turn to herbal and faith-based remedies as more affordable alternatives.

## Explanatory models of illness

A study of traditional healers’ role in the treatment of people with schizophrenia in Egypt reported that 58.19% of patients’ first consultation for their illness was with a psychiatrist, whereas 41.81% of patients consulted a traditional healer first.^13^ Those who consulted a psychiatrist believed their symptoms were caused by mental illness, whereas those who sought traditional care methods had attributed their symptoms to other causes: 21.55% believed they were possessed by jinn, 8.19% believed the illness to be a result of envy and 12.07% thought black magic was being used on them. Illness has been culturally attributed as one of the possible repercussions that might emerge from being under the curse of the ‘evil eye’, typically inflicted out of jealousy or envy. In 2010 a household survey conducted in Saudi Arabia reported that approximately 42% of participants were referred to traditional healers for healthcare and advice.^14^ Common complaints for seeking care and presenting for treatment included physical symptoms such as abdominal pain, flatulence, low back pain and headaches, as well as psychological symptoms such as low mood and depression. Patients sought out traditional medicine for a variety of reasons, including faith in the efficacy of the therapy, a preference for natural and organic remedies and resistance to attempted medical interventions. Healing techniques usually consisted of reciting the Holy Quran, herbal medicines, cautery (the process of burning a body part to close a wound) and cupping (the practice of applying heated cups to the skin).^14^

For millennia Arabic healers have offered a variety of herbal, spiritual and other physical treatments for challenging illnesses such as male infertility and impotence in the neighbouring Arab Gulf and Levant nations of Lebanon, Syria, Palestine and Jordan.^5^ Throughout history, it became known that Middle Eastern ethnomedical views on the origins of illness and how to treat it are nuanced and complicated, refusing simple classification.^5^ Nonetheless, views regarding illness range from naturalistic and physical causes to personality and social factors, to supernatural and spiritual ones. For example, in the Middle East, Egyptian ethnomedical beliefs about the causes of infertility range from humidity to sorcery and include the possibility of an ‘open back’, a shock, a ‘polluting entrance’, an enraged spirit under the ground and God's will.^5^

## Fusing traditional and Western models

Any sense of cognitive alienation toward treatments that were first promoted by non-Arab physicians was eliminated by the historical engagement of Arabs in the formation of the Western biomedical paradigm and its subsequent reintroduction into the Arab world.^4^ Many healthcare philosophies have considered the advantages of fusing traditional and contemporary treatment after the World Health Organization recognised the significance of traditional medicine.^15^ According to some groups, this integration will result in a more holistic approach to healthcare, focusing on the mental, spiritual, emotional and physical well-being of patients. Since spirituality is a strongly embedded concept in the Arab World, this integration can ensure meeting patients’ spiritual and physical needs.^16^ This is especially relevant since an increasing amount of literature in Western nations centres on the connection between spirituality and illness.^16^ One study that combined medical and spiritual treatment in coronary care units in the Gaza Strip revealed cardiac patients’ opinions of the value of spiritual and mental healthcare as fundamental services. This integration is expected to have a positive impact on patient health outcomes by decreasing stress, the duration of hospital admissions and treatment fees.^16^ Enough research has been produced on the unconnected effectiveness of Western medical models versus healing traditions.^4,17^ Further research is needed on the holistic and integrative approach to medicine and spirituality in the Arab world. This is especially necessary since traditional healing techniques are already being practised in the Arab world without adequate documentation.

## Data Availability

Data availability is not applicable to this article as no new data were created or analysed in this study.
